# The ‘Sanctuary Gap’: Reviewing the Research on Captive Wildlife Sanctuary Tourism

**DOI:** 10.3390/ani15040496

**Published:** 2025-02-10

**Authors:** Siobhan I. M. Speiran

**Affiliations:** Faculty of Environmental & Urban Change, York University, Toronto, ON M3J 1P3, Canada; speiras@yorku.ca

**Keywords:** wildlife sanctuary tourism, captive wildlife tourism, wildlife conservation, animal welfare, conservation–welfare nexus, multispecies community

## Abstract

Wildlife sanctuaries have gained popularity in recent years as settings for research into human–animal relations, captive wildlife tourism, and rehabilitation. While scholars from animal studies, ethics, geography, and ethnography disciplines have turned their attention to sanctuaries, there is still limited engagement from the fields of tourism, conservation, and animal welfare sciences. Adopting an interdisciplinary approach, this paper addresses the ‘sanctuary gap’ by offering a synthesis of the existing research related to wildlife sanctuary tourism. To this end, the paper suggests preliminary definitions for *wildlife sanctuary tourism* and *wildlife sanctuary attractions*, as well as a typology of sanctuaries along a spectrum from *greenwashed* to *just*. It aims to illuminate the shadowy presence of wildlife sanctuaries across multiple disciplines and identify areas for future research. The discussion considers how sanctuaries are well-suited to research on multispecies communities, as well as the conservation and welfare of wild animals.

## Simple Summary

Wildlife sanctuaries are increasingly popular sites for research on captive wildlife tourism and rehabilitation, as well as multispecies labour, livelihoods, and communities. Although sanctuaries are gaining attention from animal studies and ethics scholars, there is still relatively little discussion from the fields of tourism, conservation, and animal welfare sciences. This paper aims to address this ‘sanctuary gap’ by offering preliminary definitions for *wildlife sanctuary tourism*, *wildlife sanctuary attractions*, and a typology of sanctuaries along a spectrum from *greenwashed* to *just* sanctuaries. Finally, it recognizes how sanctuaries, as multispecies communities, offer vital opportunities to investigate multispecies communities and the conservation–welfare nexus.

## 1. Introduction

Wildlife tourism is intensifying and diversifying, in step with urbanization and mounting demand for proximity and encounters with wild animals [1,2]. A niche of nature-based tourism, it revolves around encounters with captive, semi-captive, or free-living non-domesticated species usually in or around a protected area [3,4]. Wildlife attractions are often characterized as consumptive (e.g., hunting, fishing, and gustatory) or non-consumptive (e.g., zoos, aquaria, and viewing free-living wildlife), with the latter representing an estimated 20–40% of international tourism [1,4]. Around 2.6 million wild individuals live in 800 zoos and aquaria across 80 countries [5]; relatively little is known about the approximately half million individuals held in captive wildlife attractions around the world which are not accredited as zoological facilities [4]. More research is needed on the conservation and welfare outcomes of animals held in wildlife tourism attractions, especially those which use animals for entertainment or permit animal–visitor interactions [4,5,6,7]. Included in this category are wildlife sanctuary attractions featuring captive wildlife. As an increasingly popular form of wildlife tourism, sanctuary attractions may be viewed as an ‘alternative’ to mass tourism and traditional zoological institutions. Emerging (though limited) scholarship suggests that sanctuaries have the potential to inform both the research and management of non-zoo, captive wildlife attractions [4,8,9,10,11]. Further empirical research is needed, however, which examines the lives of animals in wildlife sanctuaries, which is a significant gap in tourism research [12,13,14].

This paper seeks to address this ‘sanctuary gap’ through conceptual contributions, building on Ross and Leinwand’s observation that “the idea that sanctuaries can aid research is no longer a niche concept” [15]. Towards this goal, it offers an interdisciplinary synthesis of existing research related to wildlife sanctuary tourism and suggests a preliminary typology of wildlife sanctuary attractions. This synthesis is supported by a qualitative review of the literature conducted with Google Scholar, PubMed, and ScienceDirect databases using the following search terms: *animal sanctuary*, *wildlife sanctuary*, *captive animal sanctuary*, *captive wildlife sanctuary*, *wildlife rescue and rehabilitation*, *captive wildlife sanctuary tourism*, and *animal rescue tourism*. Furthermore, this paper was developed alongside mixed-method, multi-sited empirical data collection on primate sanctuary tourism in Costa Rica in 2019, the details of which are presented in other publications [16,17].

The aim of this literature review was to gather relevant, extant research on (and related to) captive wildlife sanctuary tourism published in English. Given the relative underrepresentation of this specific context in existing scholarship, the search was expanded to include publications on domestic animal sanctuaries and wildlife rehabilitation when relevant to the focus of the paper [18]. It does not attempt to thoroughly survey these related fields, however, with publications chosen for their potential insights into captive wildlife sanctuary tourism. Research that referred to ‘wildlife sanctuaries’ as protected natural areas with free-ranging wildlife was excluded from the review [18]. Ultimately, this interdisciplinary and non-exhaustive review yielded 150 publications: 125 peer-reviewed articles from 54 journals, 5 academic books, and 23 book chapters. A full list of the selected publications, organized by publication type, with focal animals and keywords included where available, is provided as Supplementary Material in Table S1.

This paper is structured as follows. First, it provides preliminary definitions for *wildlife sanctuary tourism* and *wildlife sanctuary attractions*. These two concepts are currently underdeveloped in the literature likely due to the absence of a unified theoretical framework. Second, it lays the groundwork for a typology of sanctuary attractions, distinguishing between those that genuinely enhance the welfare of wild animals and those which jeopardize it. Third, the paper identifies key gaps in sanctuary research from various academic perspectives including animal welfare, conservation science, and a range of disciplines related to human–animal studies. These gaps are explored in light of the opportunities and challenges that sanctuary tourism research faces, drawing on insights from animal-based tourism [1,2,4,12,19,20,21,22,23,24], compassionate conservation [25,26], care ethics [9,25,27,28,29,30], wildlife equity and justice [11,31,32] research. Then, this paper forwards the position that wildlife sanctuaries are uniquely positioned to advance research on the *conservation–welfare nexus*, a critical concept long debated in the conservation literature, particularly in relation to stakeholder perspectives on wildlife governance issues such as rescue, rehabilitation, reintroduction, and culling practices [28,29,33,34,35,36,37,38]. Finally, the paper examines future directions for wildlife sanctuary tourism research and offers concluding thoughts.

## 2. Defining Wildlife Sanctuary Tourism

Wildlife sanctuaries generally have an amorphous presence across multiple disciplines [12,19]. They vary widely in presentation and structure; some have traditional, closed enclosures, while others offer open or hybrid forms of captivity. Sanctuaries may be open or closed to the public, and operated by government agencies, non-profits, or as private businesses—with regulation depending on the country and location [16]. Some may specialize in one type of animal, while others serve local wildlife more generally. Sanctuary residents can vary in conservation status and origin; they may be captive or wild-born, a local or an introduced species, or transferred from another sanctuary [39]. Some sanctuaries offer environmental education and outreach to local communities, as well as generate funding and awareness through paid voluntourism programs and advocacy campaigns [17,27,40].

Sanctuary tourism lacks global regulation or consensus. The term “sanctuary” is not regulated [18] which can perpetuate ‘greenwashing’ or ‘humane-washing’ that confounds both tourists and researchers seeking to visit ethically operated sanctuaries [5,33,40]. There are likely thousands of ‘fake sanctuaries’ worldwide, a phenomenon detailed and interrogated by Winders [40]. More research is necessary to differentiate between wildlife sanctuary attractions which improve animal welfare and conservation and ‘greenwashed’ captive wildlife attractions which operate under the guise of ‘sanctuary’ [4,11,34].

The only globally recognized accrediting body, the Global Federation of Animal Sanctuaries (GFAS), is tasked with recognizing ‘true sanctuaries’ which uphold “standards of excellence” and provide humane care in a “non-exploitative environment” with “ethical policies in place” [39]. As of 2023, the GFAS membership includes 132 accredited and 83 verified sanctuaries across 18 countries, representing 35,000 animals [39]. A GFAS-accredited sanctuary prohibits commercial animal trade, unguided and disruptive tours that cause stress to animals, direct contact between animals and the public, the exhibition or relocation of animals outside their enclosures for non-essential purposes, and generally does not allow captive breeding [39]. Additionally, GFAS provides sanctuaries with guidelines to enhance sustainability through strategic planning [35]. In the context of captive wildlife tourism, both the GFAS and scholarship on the welfare of wild animals recommend a ‘hands-off approach’ that restricts close interactions between tourists and animals [36].

Wildlife sanctuary tourism can cater to those looking for ethical interactions with captive wild animals within a conservation-focused setting, but these institutions can vary in title and accreditation [4]. Facilities that offer care for wild animals may adopt titles such as a “rehabilitation center”, “rescue center”, or “sanctuary”; these terms lack regulation, however, and thus can be widely and interchangeably applied to various establishments featuring captive animals [16,33]. *Animal rescue tourism* has been identified by recent scholarship as a unique form of animal-based experience that attracts individuals interested in ethical interactions with rescued animals [37]. Broadly speaking, a wildlife *rescue centre* primarily focuses on rescuing and providing immediate veterinary care to injured or sick wild animals [16]. A wildlife *rehabilitation centre* offers short-term medical care for sick or injured wildlife, while a *sanctuary* provides long-term, lifelong care for wildlife that cannot be reintroduced into the wild. The state of a wild animal found injured, ill, in immediate danger, alone, or in an illegal captive context may be brought to the attention of a sanctuary, rescue, and/or rehabilitation centre by local community members, tourists, government, or emergency service personnel [8,16].

The rescue, rehabilitation, and reintroduction of wildlife are consecutive strategies which aim to enhance their conservation and/or welfare [38]. *Rescue* involves removing animals from harmful or threatening situations [41]. *Rehabilitation* is a managed process that focuses on helping injured, ill, displaced, or orphaned wildlife recover, with the goal of either ensuring their survival for eventual release or providing life-long care in captivity when release is not possible [38]. *Reintroduction* refers to the release of rehabilitated animals back into the wild, either in the location where they were rescued or in a more suitable habitat [42]. These three processes can occur in the same sanctuary facility or across separate entities, as defined above. Sanctuaries which reintroduce wildlife are typically located within the range of the target species, and ethical, accredited sanctuaries do not display individuals intended for reintroduction to the public [16].

Sanctuaries are idiosyncratic in their commitment to reintroduction activities, as well as how a ‘successful release’ is measured (e.g., post-release monitoring). Therefore, it is important to note that not all wildlife sanctuaries reintroduce wildlife, and of those which do, not all are successful nor ‘conservation-oriented’. For example, reintroductions may be ‘welfare-oriented’ whereby individuals are released in the hopes a more natural lifestyle will improve their welfare [16]. More research is needed on the intentions and outcomes of welfare and conservation-oriented reintroductions (*ibid*).

To improve clarity around this dilemma, this paper takes up recent scholarship to propose the following definitions: *wildlife sanctuary tourism* may be defined as a niche of captive wildlife tourism, and a *wildlife sanctuary attraction* (*WSA*) as a facility which allows visitors to view wild animals (from captive-born or free-living origins) undergoing rehabilitation and unsuitable for reintroduction [4,11,12,34,36,40]. These definitions are established based on an interdisciplinary engagement with animal welfare [1,43,44], tourism ethics [6,20,31], multispecies ethnography and geography scholarship [8,9], conservation studies focused on wildlife rehabilitation [14,45,46] and sustainability [10,47].

### The Spectrum of Sanctuary Tourism

**Figure 1:**
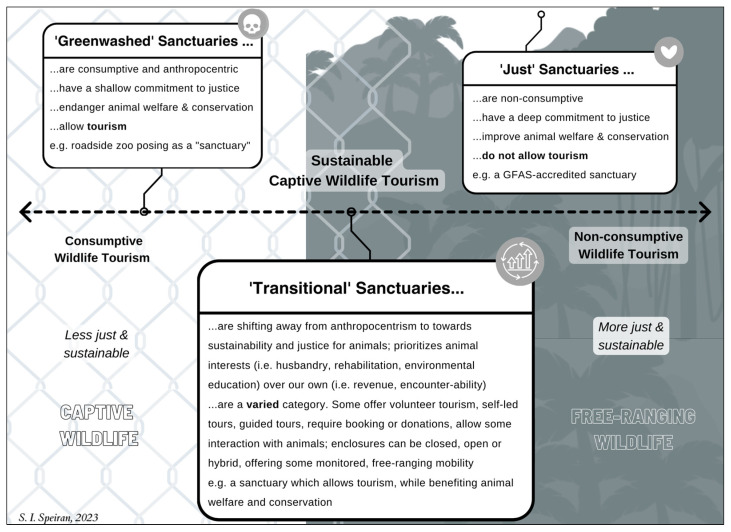
*Spectrum of Sanctuary Tourism*: from greenwashing to interspecies justice.

Grounded in existing research that differentiates sanctuary attractions from zoos, aquaria, and other forms of captive wildlife tourism [4,14,48,49], this section delves into the (in)distinctions between ‘true’ wildlife sanctuaries and greenwashed attractions [11,40,50,51]. To enhance clarity, a preliminary typology of sanctuaries which categorizes them based on their commitment to justice and sustainability is proposed for its utility [32]. This typology is illustrated along a *Spectrum of Sanctuary Tourism*, which ranges from consumptive to non-consumptive attractions (Figure 1) [21]. The goal of this typology is to contribute to ongoing efforts to improve animal welfare by adding nuance to the under-researched phenomenon of sanctuaries [6,7,10,14,21,31].

At the consumptive end of the spectrum are *greenwashed sanctuaries*. These captive wildlife attractions deceptively use the term “sanctuary” to imply a dedication to long-term care or rehabilitation of wild animals, primarily to attract tourism and generate revenue. In practice, however, they compromise animal welfare and conservation efforts and exhibit little to no concern for multispecies justice or sustainability [21,33,40,52]. The *Black Jaguar White Tiger Foundation* in Mexico is an example of such a greenwashed sanctuary. Greenwashed sanctuaries and unethical wildlife tourism transfer the responsibility of rehabilitation and rewilding onto legitimate sanctuaries [12], many of which operate through models of “custodial labor” and “commercial volunteerism” as observed in Parreñas’ study of in situ orangutan rehabilitation on Borneo [9].

*Transitional sanctuaries*, situated in the centre of the spectrum, fund their operations, rehabilitation work, and other activities through paid tourism opportunities that genuinely enhance the welfare of the animals in their care and support conservation initiatives. These sanctuaries combine the conservation-oriented aspects of modern zoos with the welfare-focused, rehabilitative goals of wildlife medicine, offering a sustainable form of captive wildlife tourism [11,34,53]. Wildlife may either be free-living following reintroduction into the wild, or live in (semi-)captive enclosures. *Rescate Wildlife Rescue Center* in Costa Rica and *Elephant Nature Park* in Thailand serve as examples of transitional sanctuaries. Transitional sanctuaries committed to systems of high welfare and conservation outcomes (prioritizing this nexus over profit) may improve the lives of wild animals in captivity and the wild, as well as achieve sustainability and justice for wild animals [4,10,11].

On the non-consumptive end of the spectrum are *just sanctuaries* with a deep committed to animal justice and sustainability. While both just and transitional sanctuaries aim to support animals impacted by human activities through caregiving—which includes reintroduction into the wild and/or lifelong care in captivity—just sanctuaries do not allow wildlife tours or public viewing of resident wildlife. This places them outside the realm of wildlife tourism, represented in Figure 1 as unattached to the tourism spectrum [10,11,31,51]. Wildlife can be free-living following reintroduction into the wild or housed in semi-captive enclosures designed to replicate natural, free-living conditions as closely as possible. *Born Free USA Primate Sanctuary* exemplifies a just sanctuary.

Just sanctuaries are not open to the public but can still engage meaningfully in community outreach and environmental education through public events, school programs, citizen science initiatives, and the sharing of the ‘behind-the-scenes’ of sanctuary operations through social media and/or press engagement. For example, the *Vervet Monkey Foundation* (https://vervet.za.org/wp-content/uploads/2024/02/Annual-Report-2023.pdf, accessed on 21 January 2025) in South Africa won the Outstanding Wildlife Sanctuary Award from the GFAS in 2023 for their commitment to the rehabilitation and reintroduction of vervets. While the sanctuary is not open to the public, it accepts volunteers and encourages environmental stewardship amongst the local community and schools to address human–wildlife conflict through the promotion of coexistence.

Malawi’s only wildlife sanctuary, *Lilongwe Wildlife Centre* (https://lilongwewildlife.org/news/award/, accessed on 21 January 2025), received the Outstanding International Sanctuary Award from GFAS in 2023. While wildlife undergoing rehabilitation are not viewable by the public, the sanctuary welcomes visitors on-site to birdwatch or explore their scenic trails, forests, wildlife sculptures, café, and gift shop– which contribute to funding the rehabilitation work. The site also features a conference room and environmental education centre. One may ‘symbolically adopt’ a pangolin, the most trafficked wild animal in the world, which functions as a donation for which an information ‘pack’ and certificate are provided, as well as images of the pangolin throughout its adoption. These are just some examples of the creative opportunities employed by sanctuaries to generate interest and concern for resident wildlife and conservation at large.

In the Costa Rican context, wildlife facilities with individuals intended for reintroduction are non-profit and closed to the public and licensed as ‘rescue centres’. Facilities with non-releasable wildlife may offer public viewing if wildlife are displayed in an educational manner with adherence to good welfare practices, licensed as ‘zoos’. Costa Rican wildlife facilities may be dually-licensed as both a rescue center (i.e., private) and a zoo (i.e., public-facing), while advertised collectively as a ‘sanctuary’.

One of the major known causes of wildlife rescue in Costa Rica is electrocution from uninsulated power lines and transformers [17]. Consequently, many sanctuaries raise funding for preventative measures, including the insulation of these hazards and the establishment of wildlife corridors (e.g., hanging ‘rope bridges’) to connect fragmented habitats and provide an alternative, safe crossing for arboreal species that use power lines to traverse roadways and urban centres. Sanctuaries which allow tourism, in tandem with conservation-based outreach and advocacy, are examples of transitional sanctuaries. It is important to note, however, that the phenomenon of voluntourism complicates tourism research on just wildlife sanctuaries since they may allow short-term volunteer stays which differ little in practice from traditional tourism. More research is needed to determine the difference in the impact of voluntourism on wildlife tourism.

The concept of just sanctuaries may seem aspirational, though sanctuaries which seek to “counter a world of enclosure” undoubtedly exist [54]. Just sanctuaries may undo processes of objectification, producing novel ‘wild’ subjectivities that defy neat scientific categorization; many wild animals in tourism exist “between wild and tame” [55]. In these transspecies borderlands, sanctuary animals can be “reconceptualized as beings possessing subjectivity, agency, and intentionality, active in configuring both the environment they inhabit and their interactions with people” [56] (p. 681).

The practices of just and transitional sanctuaries may offer a “new starting point: for conversation, imagination, and new relations” [51]; they are well-positioned to host multispecies research “guided by respect for otherness, geared to ensuring animals’ flourishing and committed to a nonviolent ethic” [57,58] (p. 185). According to Blattner et al., sanctuaries can support “rich and meaningful forms of animal agency, including forms of relational agency that might only be possible in a multispecies community that includes humans”, whose “involvement may support unique forms of agency” [59] (p. 17). This is one step towards enfranchising animals as stakeholders in wildlife tourism [53].

On a case by case basis, sanctuaries may provide good welfare and conservation outcomes, multispecies livelihoods, shallow or even intermediate levels of justice for involved species [4,10,11,24]. Futhermore, by combining modern zoos’ conservation mandate with the welfare and care-based objectives of wildlife medicine and rehabilitation (i.e., the conservation–welfare nexus), just (and some transitional) sanctuaries can foster varying degrees of interspecies justice and sustainability. The next section examines and synthesizes literature related to the captive wildlife sanctuary context, drawing from both animal welfare and conservation sciences, as well as from animal studies research across multiple social science and humanities disciplines.

## 3. Gaps in the Study of Wildlife Sanctuary Tourism

### 3.1. Perspectives from Wild Animal Welfare Research

Humans have a myriad of impacts on the welfare of both captive and free-living wild animals, yet most welfare research has focused on agricultural, laboratory, and companion animal species [60,61]. In a general sense, the welfare of an animal refers to how it experiences its life; it is holistically measured through a combination of biological functioning (i.e., health), mental experience (i.e., affective state, feelings), and the naturalness of its living conditions. Accredited zoos, aquariums, and animal industries worldwide base their management and use of animals on the Five Domains of the animal welfare paradigm [44]. Since its introduction three decades ago, the Five Domains model for animal welfare assessments has been continuously updated [44]. Originally focused on animals’ experience of pain, distress, fear, anxiety, thirst, and hunger as indicators of their welfare, in its 2020 update, the Five Domains have evolved to integrate human–animal interactions and animals’ positive affective states (e.g., pleasure, comfort, choice, control, agency, etc.) as indicators of their welfare [44].

Across the research on wild animal welfare, sanctuaries are not favoured as locations for research with wild animals; for example, a bibliometric analysis of over 1000 publications on wild animal welfare between 1966 and 2007 found that only 10 studies were conducted in a sanctuary context [62]. Most wild animal welfare studies surveyed by Goulart et al. focused primarily on health and ex situ conservation as indicators of animal welfare, with only a minority of studies conducted in the wild (~10%), a third of which investigated animal welfare based on in situ conservation activities [62]. Additionally, the majority of studies were theoretical (~58%) rather than experimental, and most were published in English and conducted in Europe and North America. Over 90% of the theoretical research centered on ethics, while experimental studies mainly focused on environmental enrichment or ex situ conservation efforts [62]. The definitions of ‘theoretical’ and ‘experimental’ employed in this study, however, are not entirely clear, and it is uncertain how observational studies of animal welfare were classified.

The study of wild animal welfare is fraught with both ethical and practical challenges. Ethical concerns about manipulating experimental variables in zoo settings may partly explain why much of the research is theoretical. Nevertheless, research in this field appears to be growing. There is a need for more studies from species-rich, developing countries, as Central and South America, for example, account for only 2% of publications on the topic [62]. While there is limited research on the welfare of animals in free-living contexts [63], protocols for assessing the welfare of free-roaming, semi-feral, and working equids in development [64,65,66,67] and wildlife market contexts have been proposed [68].

Animal welfare is frequently overlooked when investigating the lives of animals in tourism contexts. Assessing an animal’s welfare status requires a holistic approach rather than relying solely on indirect indicators (e.g., tourist satisfaction, *TripAdvisor* reviews, or success in rehabilitation or reintroduction), which, while useful, should only serve as a starting point [7,14]. Frameworks for assessing the welfare of captive wild animals in zoos can be applied to other captive attractions, such as sanctuaries, though with varying success [69,70,71,72,73]. Models have been developed to enable tourists to self-assess attractions based on their commitment to welfare, conservation, governance, and animal-informed consent [7,14,74]. There is an evidenced need to improve animal welfare literacy amongst researchers, practitioners, and tourists to achieve more proximate, applicable solutions to improve the conditions under which animal tourism occurs [75,76,77].

Commenting on the nexus between animal welfare, the environment, and sustainability, a 2023 report from the *World Federation for Animals* stated that a ‘high welfare system’ contributes to “multiple dimensions of sustainable development” and is essential to “effective adaptation” for climate change and to achieve some of the *UN’s Sustainable Development Goals*, as animal welfare improves and safeguards biodiversity [47]. A growing body of the literature surrounding the impacts of tourism activities on the welfare of wild animals has accumulated over the past decade [1,5,6,14]. A large-scale audit of the types of animal–visitor interactions available in facilities accredited by the *World Association of Zoos and Aquaria* found that most allowed direct animal interaction, such as feeding or touch encounters [5]. A similar audit to collect in situ data on sanctuary attractions is recommended and could follow the protocol outlined by Fennell et al. and de Mori et al. [14,69].

A sweeping, although ex situ survey by Moorhouse et al. of global (non-zoo and non-hunting) wildlife tourism attractions found that most have a negative impact on the welfare and conservation status of the animals featured [4]. The sanctuary attractions they surveyed, however, were an important exception, with positive impacts for involved species [4]. Nevertheless, sanctuary attractions vary in their institutional commitments to animal welfare, conservation, and tourism management: three factors which are challenging to reconcile with tourism practice [14,78]. From these findings and other scholarship on the subject, there is a definite need for empirical research to identify, explore, and validate in situ how sanctuary attractions contend with the interdependence of animal welfare and conservation in practice [4,10,11,17,34].

Amongst the increasing evidence emerging around the potential for sanctuary attractions to improve the welfare and conservation outcomes of animals involved, a novel content analysis examined the images tourists share on social media after visiting wildlife attractions [36]. Employing a qualitative, ‘netnographic’ method to compare images from captive, non-zoo attractions with those from sanctuaries, the researchers used the conservation and welfare outcomes identified by Moorhouse et al. associated with these attractions [4,36]. While tourists who visited captive, non-zoo attractions frequently posted selfies with the animals, post-visit photos from sanctuary tourists most often featured the animals alone (ibid). Through content analysis of the images and captions shared, it was determined that sanctuary tourists demonstrated more ecocentric values and a higher level of involvement (i.e., sharing knowledge of animals, environment, conservation, and action-oriented behaviour), which correlated to Moorhouse et al.’s findings that the sanctuaries sampled had positive welfare and conservation outcomes. In contrast, all captive, non-zoo attractions led to negative welfare and conservation outcomes for focal species, and content analysis of post-visit photos reflected these unethical practices and anthropocentric standpoints. The authors conclude that providing good conservation and animal welfare outcomes for wildlife appears to influence human behaviour and perspectives [4,36].

Given the lack of global wildlife tourism regulations, wildlife selfies (sometimes called ‘photo-prop tourism’) are not uncommon amongst so-called wildlife sanctuaries, which may advertise using these images to attract visitors and encourage empathy and interest in conservation. Evidence suggests that sharing images on social media which depict humans holding and/or posing in close proximity to wild animals (i.e., “wildlife selfies”) appears to influence desirability for close encounters amongst wildlife tourists and/or encourage ‘exotic pet’ ownership [50,79]. A review of the literature surveying the influence of social media on public perceptions of wild animals found that, while it can be a valuable tool for sharing information about ethical human–animal relations, social media is also a virtual space with material consequences for animals’ lives and bodies exploited by the wildlife trade and unethical encounters [50]. Furthermore, a review of the literature on anthropomorphized representations of animals in contemporary mass media found that such depictions influence the public’s perception of animals’ natural behaviours and conservation status and influence their desirability as companion species [80]. Finally, a quantitative content analysis which coded and analyzed 670 wildlife images posted on the Instagram accounts of 160 conservation organizations found that most images featured the animal without human presence, which can still encourage support for conservation efforts [81]. Conservation organizations aiming to boost engagement with their social media content are advised that images of humans interacting with wild animals are not necessary to foster such engagement and can impart the wrong message about what constitutes ethical human–wild animal interactions [81].

### 3.2. Perspectives from Interdisciplinary Animal Studies

The academic fields of multispecies ethnography [45,59], animal geography [8,54,57,58], and animal-based tourism studies [24,82] have contributed to a deeper understanding of sanctuaries. This section draws from these disciplines, as well as from research on wildlife tourism, which forwards ethics of care and kinship [28,83,84], wildlife equity and justice [6,10,11,31], and compassion-based practices [25,43,61].

The lives, labours, and livelihoods of animals are of integral importance to global conservation and tourism activities. Still, consideration for the ethics of animal use within these intertwined practices, writ large, appears anthropocentric and isolationist; within the academic field of tourism studies, animal ethics is “virtually terra incognita” [85]. In addition, most tourism research has focused on a limited range of taxa, such as wildlife in captivity or easily visible in large groups [86].

Despite a demonstrable interest in ecocentrism, animal rights, and ecofeminism, the tourism literature has engaged less with animal welfare as a branch of science than it has with the ethical position of animal welfare, especially in assessing the state of animals as they attempt to cope within contrived environments [13,47,60,61]. Research from Winter reviewed seventy-four articles on animal ethics, welfare, and tourism in ten tourism journals and identified a spectrum of ethical positions, including animal rights, ecofeminism, animal welfare, ecocentrism, utilitarianism, and instrumentalism [2]. Winter concludes that while most of these articles challenge animal use in tourism for entertainment purposes, there is a need to apply “specific ethical principles to tourism situations involving animals” and develop more research on the “unique” relationships humans and nonhumans form during tourism encounters (p. 18) [2].

Sanctuaries may have the potential to enhance the conservation status of sanctuary species by fostering environmental literacy among tourists and the local community, particularly in understanding the repercussions of human activity on animal welfare and conservation efforts [87,88]. Thompsen et al. sampled twelve sites of wildlife ecotourism across four countries, which included five wolf sanctuaries in the USA and two wildlife sanctuaries in Costa Rica [10,34]. In Costa Rica, wildlife sanctuaries can be NGOs or privately owned ventures. Still, they must have legal accreditation as zoos if they seek to present captive wildlife to the public, which is mandated in a naturalistic, non-interactive, and environmentally educational way [10,16,17]. They found that “sanctuary models operated mixed-access facilities in which there was a sanctuary side—open to ecotourists, and a rehabilitation side—closed to the public” [10] (p. 11). Thomsen et al. also posited two key themes about the sanctuaries they sampled as models for wildlife ecotourism: that they “emphasized biodiversity conservation and engaged in educational outreach”, and “actively promoted welfare of nonhuman animals” [10] (p. 10). These findings are consistent with conclusions from Speiran, who visited eight wildlife sanctuaries in Costa Rica to investigate primate welfare, conservation, and interspecies labour at these sanctuaries [17].

The phenomenon of wildlife sanctuaries as tourist attractions has also gained traction among scholars adopting a relational ethic of care or compassion [17,25,28,82] and those examining their potential to offer a humane, ethical approach to conservation, tourism, or both [8,10,15,40]. Still, they have received limited attention from tourism, conservation, and animal welfare scholarship. These historically anthropocentric disciplines are encouraged to centre the animal experience with attention paid to animals’ emotions, positionalities, perspectives, and their relationships with humans [45,57,58]. In doing so, they may draw from studies which investigate the potential of sanctuaries to restore wild animal agency through the decolonizing and decommodifying labours of transspecies caregiving and animal advocacy [8,9,17,27,30,55,59,89,90].

More research is necessary to determine how wildlife sanctuary tourism may reinforce or disrupt the socio-ecological inequalities stemming from the colonial relations associated with captive wildlife management and tourism. For example, while contemporary zoo and safari tourism may claim to support conservation, both are tied to colonial legacies of displacement and exclusion. In brief, during the nineteenth and early twentieth centuries, the colonial powers of Europe arranged the import and exhibition of enslaved humans and nonhumans from the colonial territories; their constructed ‘exotic’ Otherness contrasted with the perceived urbanity of European society [91,92]. This fascination with ‘human zoos’ spurred the development of Western international tourism industries [92]. Relatedly, safari tourism is frequently associated with ‘fortress conservation’. The latter describes a colonial conservation paradigm in which a protected natural area is boundaried to ‘preserve’ wild species while restricting the presence of Indigenous communities and traditional use of the area’s natural resources [90,91,92]. As Anderson observes, “zoos ultimately tell us stories about boundary-making activities on the part of humans” [91]. The same may be said of sanctuaries, which make decisions around which rehabilitated animals have recovered enough of their ‘wild autonomy’ to be successfully reintroduced and which require life-long support in captivity.

Investigating whether wildlife sanctuaries inherit these intersectional inequalities from the colonial history of zoos and fortress conservation-style tourism should be considered on a case-by-case basis with cultural and historical sensitivity. Sanctuaries in developing contexts may be owned, managed, or staffed by expatriates and transnational ‘volontourists’. Thus, future research could investigate the neocolonial relations of transnational, cross-species care in the sanctuary context. Likewise, it is pertinent to consider the decolonizing potential of certain sanctuaries to care for rescued wildlife and potentially restore them to their habitat (as described henceforth), as well as provide socio-economic benefit to the host community through employment and/or outreach.

In *Decolonizing Extinction,* Parreñas’ multispecies ethnography of orangutan rehabilitation centers in Borneo offers a provocative insight: “the moral weight of extinction is significant enough to generate an industry of volunteer tourism for threatened wildlife” [9]. Engaging in participant observation at a wildlife rehabilitation center in Guatemala, animal geographer Collard observed in her research that to rebuild the wild lives of rescued individuals, the center must “put animals back together and take commodities apart”, such that they “divest them of their dependence on human inputs and of their petlike behaviors” [8]. Speiran explores similar findings in a case study of sanctuary monkeys in Costa Rica, positing that the process of wildlife rescue, rehabilitation, and reintroduction is also a process of de-commodification, signifying the restoration of agency, which prepares wild animals to return to their ecological roles in the wild (i.e., ecosystem services) [17].

There is no single form of tourism or ethical framework suited to every situation [12]. In cultural contexts where adopting a liberationist approach to wildlife tourism is impractical due to economic, temporal, spatial, and social limitations, responsibly managed sanctuaries may offer a viable solution in developing regions where less anthropocentric frameworks are not realistically feasible [93]. For example, studies have observed a “work-for-care cycle” in Thai elephant “sanctuaries”, which are considered more ethical, and elephant “camps”, which are considered less ethical [3,28,93,94,95]. In both captive elephant tourism contexts, the work-for-care model can be criticized as ethically questionable since elephants are generating economic capital, even if this labour funds their care [93]. In Thailand, almost all elephants are part of the tourism industry, most living in the ~50 camps located around the country [3].

Relational, ecofeminist ethics of care and a less anthropocentric animal welfare perspective endorses the sanctuary model of elephant tourism in Thai contexts and can serve as guiding ethics for their operation [3,93]. There is evidence of some Thai elephant camps encouraging captive wildlife tourism practices towards an ecocentric ethic, described as “a paradigm shifter” for elephant welfare [94]. Observing a shift in management practices over 15 years in select Thai elephant camps away from inhumane practices (e.g., riding, negative reinforcement) to meet the physiological, psychological, social, and nutritional requirements of elephants, Bansiddhi et al. write the following: “It is our hope that new generations of mahouts and elephants will result in good management for elephants and enriching, educational experiences for tourists” and call for a scientifically evidenced set of guidelines and enforceable regulations for tourist elephants in Thailand [94] (p. 172).

Notwithstanding the cultural context in which these attractions exist, certain establishments such as some ‘zoos’, camps, and other wildlife-focused venues may, in fact, count as wildlife sanctuary attractions, even if they do not label themselves as such [6]. For example, in Costa Rica, it is illegal to have wild animals in private collections, and only zoos can display wildlife to the public. Facilities with captive wildlife are legally designated as conservation-mandated zoos, breeding, or rescue centres [10,17]. Many of these facilities, however, self-identify as, or associate with, the term sanctuary; for example, three facilities in Costa Rica are nationally accredited as zoos and rescue centers and accredited by the GFAS, as opposed to a zoological accrediting body [16].

Increasing scholarship considers the ethical dilemmas implicit in wildlife conservation and care [21,96,97,98]. Future research could explore why some non-zoo, captive wildlife tourism attractions self-identify as particular facilities (i.e., wildlife sanctuaries, farms, camps, rescues); see also [32,51]. As Hooper recommends, to address the prevalence of poor animal welfare across the wildlife tourism industry, accredited zoos in a range of countries and their counterparts in colonial nations abroad should collaborate to generate a unified, holistic practice (i.e., a “global cooperative of zoos”) which supports non-accredited zoos in range countries to meet high standards, while also holding each other accountable since, as aforementioned, accredited zoos do not always meet their obligations to high animal welfare and/or conservation practice [90]. This is a critical step towards “decolonising animal tourism from within” [90]. Therefore, non-accredited zoos and/or sanctuary attractions should be supported by their accredited colleagues in pursuit of positive outcomes for the conservation and welfare of involved species, as well as sustainable tourism practices.

## 4. Discussion

### 4.1. Sanctuaries and the Conservation–Welfare Nexus

High animal welfare is integral to biodiversity and conservation [47]. Thus, sanctuaries conducting species reintroductions and tourism encounters with rehabilitated wildlife must consider the nexus of welfare and socio-ecological system integrity as it influences the sustainability of their operation [47]. Sanctuaries are well-positioned for research on this ‘conservation–welfare nexus’, a relatively understudied topic within animal-based tourism [12].

In a rare discussion of the conservation–welfare nexus in the context of animal-based tourism, von Essen et al. comment with the following: “The thrust of this dilemma is a trade-off between individual welfare of sentient beings and species level welfare, often conceptualized as a conflict between the sentientistic and ecocentric” [20]. Tensions arise when these two somewhat oppositional ethics are applied to wildlife tourism, with the interests of human stakeholders balanced against the interests of individual animals (i.e., welfare) and the species at large (i.e., conservation). While an ecocentric ethic prioritizes the interests of wild populations, species, and the ecosystem at large, an animal rights perspective prioritizes animals as ‘subjects of a life’, with humans unjustified in killing or causing animals harm [99]. It is critical to develop literacy around the conservation–welfare nexus for scholars and practitioners mitigating hard choices and trade-offs in wildlife sanctuary tourism.

The ongoing dialogue surrounding the conservation–welfare nexus has given rise to various positions in the fields of animal ethics, conservation, and welfare [60]. These include compassionate conservation [26], conservation welfare [43], a ‘practical ethic’ [61], and the ‘duty of care’ [25,29]. This paper proposes that sanctuary attractions adopt a hybrid ethical framework, as suggested by Learmonth, who has compared these approaches [25]. This perspective is supported by Beausoleil, a self-identified “compassionate conservation welfare scientist” who has published in animal welfare and co-authored the 2020 update to the Five Domains of Animal Welfare [44]. Beausoleil cautions that neither compassionate conservation nor conservation welfare is “faultless or sufficient” to address the wide range of concerns regarding nature conservation (p. 12) [44]. Conservation welfare is, however, promoted as a more permissive ethical framework which mandates consideration for individual wild animal welfare amongst conservation biologists and policy-makers [43,100].

While conservation welfare has limitations stemming from its welfarist (and thus utilitarian) ethical foundation, “it is clear about which animals are to be considered […], what harm/suffering are and how they can be assessed, and how to fulfil our obligations to consider animal welfare as part of contextualized decision-making, i.e., by minimizing harms whenever possible” [100] (p. 13). Drawing from animal welfare science, conservation welfare integrates evidence-based consideration of individual animals into conservation efforts, such as by attending to their affective experience and mental state with the intention of minimizing suffering and encouraging compassion [100]. For its moral consideration of individual animals in the context of conservation management, a conservation welfare approach is encouraged for both researchers and practitioners of wildlife sanctuary tourism.

To improve outcomes along the conservation–welfare nexus in sanctuary settings, the following suggestions are made. Welfare assessments, which have long been used to guide practices in zoos and aquaria, should be integrated into research and practice with sanctuaries [14,69,71]. Sanctuaries (both accredited and unaccredited) should aim to provide good lives for wild animals in their care based on a holistic vision of welfare which accounts for environmental enrichment, physical health, mental experiences, cognitive bias, agency, choices, and relations to keepers and tourists [12]. In tandem, sanctuaries should positively contribute to wildlife conservation through meaningful activities that safeguard biological diversity, species, and ecosystem integrity [12]. This may include wildlife rehabilitation and reintroduction, socio-economic benefits to the local community, environmental education and outreach with local and tourist groups, and the implementation of wildlife corridors and reforestation projects [12,17,28,101].

### 4.2. Sanctuaries as a Multispecies Community

From a socio-political perspective, sanctuaries can be viewed as places which empower rescued wild animals to configure their own social structures rather than be paternalistically relegated to the roles of victims or refugees. More investigation into developing standards for wild animal use in tourism is necessary, especially that which recognizes wildlife as co-shapers of relations and reality in tourism encounters [102]. As argued in Speiran and Hovorka, space must be made for the deliberation of animal governance in a way which is mindful and inclusive of animal interests [12]. Regulation and policy-making must recognize animals as stakeholders in decisions which affect the integrity of their welfare as individuals and conservation as a species [12,57,58,103]. This necessitates redefining the boundaries of moral consideration, which have historically excluded animals from political discourse [12,26,32]. In tourism contexts, this requires decentering the interests of tourists and engaging with animals’ perspectives and interests, asking what constitutes a “luxurious life” in captivity, in which wild animals “thrive rather than just survive” [104] (p. 579).

Wildlife tourism research and practice should integrate justice frameworks which function on precautionary principles and an anti-discrimination approach to shift the responsibility for responsible animal care from advocacy groups onto the tourism operators. An interdisciplinary investigation into wildlife sanctuary attractions as places of interspecies justice determined that they can only offer “intermediate justice” at best and cannot provide “deep justice” [11]. This may foster the development of a care-*full* multispecies community in sanctuary [12,28,59]. In this way, sanctuaries may shift the paradigm for wildlife tourism from an “animal space” of human control to a “beastly place” of animal sovereignty [56] to envision and transform the captive, non-zoo wildlife tourism industry to ‘bring the animals back in’ to tourism practice in a way which supports their agency [6,12,59]. In summary, this paper has posited that some sanctuaries have the potential to facilitate making nonhuman, non-natal kin in the Anthropocene [51,83,84] and offer shallow to intermediate levels of justice for animals [10,11,17] through transspecies care work [30,45] and a commitment to ethical operating standards [10,47].

The increasing scholarship supports the notion of sanctuaries as sites of interspecies labour [17,105], decommodification [8], and decolonization [9], which emerge through the practice of wildlife rehabilitation. As described in Speiran and Hovorka [12], when wild animals are fixed into commodities or laborers for the benefit of humans, “processes of severing” take place between animals and their “ecological, familial, and social communities” [54,106,107]. These severed connections are replaced by human systems of management and husbandry [8]. The conservation-oriented mandate of reintroduction “endeavors to retie the severed connections that comprise the animal’s former ecological niche” and lifeworld [12,16,42,108,109,110]. Some sanctuaries may allow those individuals who cannot be reintroduced or who struggle to adjust to non-natal kin in captivity to access agency and political voice through the promotion of a multispecies community where unique, unruly ways of ‘being animal’ coalesce to generate novel ways of being *with* [8,55,57,58,59,111,112]. Future research could explore how sanctuaries—as a multispecies borderland [56], commons [113], or political demos [51,112]—can support “rich and meaningful forms of animal agency, including forms of relational agency that might only be possible in a multispecies community that includes humans” [59] (p. 17).

There is a need for imaginative, potentially disruptive methods of communicating with animals on their own terms and incorporating their interests into our decision-making spaces [84]. Storying the lives of individual animals entangled in tourism economies can illustrate the meaning of ‘animal personhood’ in this context and how we might recognize our relations with them in research and practice [31]. Forwarding ‘kinmaking’ as a “critico-creative, disruptive space and fitting thoughtscape for transitioning into more-than-tourism (studies)”, Pernecky encourages “imagin[ing] that the mass tourism structures and systems of ordering are superseded by relational mobilities, relational hospitality, and ways of being in and with places, peoples, and nonhumans that are non-destructive and critically hopeful” [84]. Put simply, sanctuaries should be a multispecies forum.

## 5. Conclusions

In conclusion, this review of the literature has highlighted that wildlife sanctuary tourism faces many of the same limitations as wildlife rehabilitation efforts and animal-based tourism more broadly. This includes the challenge of cultural relativism in developing and applying global regulations to local wildlife tourism practices. Until the gap is bridged between the global regulation of wildlife tourism and local practices, no one form of tourism or ethical framework suits every cultural context. Responsibly managed sanctuaries, however, may be a local solution for nations where less anthropocentric frameworks are not realistically achievable at present.

As a step forward, this paper has offered a typology of sanctuary attractions, from ‘fake’ *greenwashed* sanctuaries to *transitional* and *just* sanctuaries. Just (and some transitional) sanctuary attractions prioritize interspecies care and wildlife advocacy and are thus ideal settings for research which employs a hybrid, relational approach and participatory, multispecies methods. Such sanctuaries pursue positive outcomes along the conservation–welfare nexus and affirm the agency and interests of wild animals in their care. Thus, they may offer a place for researchers and practitioners to envision a more sustainable, ethically robust paradigm for wildlife tourism and multispecies relations.

This paper has underscored the necessity of continued empirical research into how sanctuary attractions can support the welfare and conservation of wild animals while promoting environmental literacy and ethical tourism practices. There is especially a pressing need to enhance welfare literacy amongst tourism stakeholders—researchers, practitioners, and tourists alike. Future investigations into sanctuary tourism attractions should also consider relational ethics of care, which prioritize the well-being of animals and/or recognize their agency. A justice-oriented, precautionary approach to the regulation of wildlife sanctuary tourism is crucial to ensure animals’ interests are accounted for in decision-making, which impacts the socio-ecological system of which they are a part.

By regarding a wildlife sanctuary as a multispecies community where animals’ interests and political voices are taken seriously, we can begin to imagine a more inclusive form of wildlife governance which recognizes animals as active stakeholders in the decisions which affect their livelihoods and conservation. While sanctuary tourism may not be a universally applicable panacea [12], it may serve as a departure point for a collaborative, inclusive approach to captive wildlife tourism, which shifts animals from the margins of our consideration to the forefront [10,31,45,58,100].

## Data Availability

The original contributions presented in the study are included in the article, further inquiries can be directed to the corresponding author.

## Supplementary Materials

The following supporting information can be downloaded at: https://www.mdpi.com/article/10.3390/ani15040496/s1, Table S1: Literature Review on Wildlife Sanctuaries.
