# Supplementary material for: The ‘Sanctuary Gap’: Reviewing the Research on Captive Wildlife Sanctuary Tourism
Source: Animals (Basel). 2025 Feb 10;15(4):496. doi: 10.3390/ani15040496 (PMC11851909; doi:10.3390/ani15040496)
Supplement: Supplementary file 1 [file animals-15-00496-s001.zip › animals-3342631-supplementary.pdf]

#	Sorted: alphabetically by journal title	Literature Review on Wildlife Sanctuaries<br>**Search Terms**: animal sanctuary, wildlife sanctuary, captive animal sanctuary, captive wildlife sanctuary, wildlife rescue and rehabilitation, captive wildlife sanctuary tourism, animal rescue tourism	**Search Engines**: Google Scholar, PubMed, ScienceDirect	
		**Books, Chapters & Other**		
	**Type of Publication**	**Citation**	**Keywords**	**Focal Animal(s)**
1	Book	Abrell, E. (2021). Saving animals: Multispecies ecologies of rescue and care. U of Minnesota Press.	n/a	Animals (generally)
2	Book	Cuny, L. M. (2010). Through Animals' Eyes: True Stories from a Wildlife Sanctuary. University of North Texas Press.	n/a	Wild Animals (generally)
3	Book	Giroux, V., Voigt, K., & Pepper, A. (Eds.). (2023). The Ethics of Animal Shelters. Oxford University Press.	n/a	Animals (generally)
4	Book	Palmer, A. (2020). Ethical debates in orangutan conservation. Routledge.	n/a	Primates (Orangutans)
5	Book	Parreñas, J. S. (2018). Decolonizing extinction: The work of care in orangutan rehabilitation. Duke University Press.	n/a	Primates (Orangutans)
6	Chapter	Abrell, E. (2019). Animal sanctuaries. In The routledge handbook of animal ethics (pp. 569-577). Routledge.	n/a	Wild Animals (generally)
7	Chapter	Alexander, S. D., Waters, S., Aldrich, B. C., Shanee, S., Clarke, T. A., Radford, L., ... & Dempsey, A. (2023). The Past, Present, and Future of the Primate Pet Trade. In Primates in Anthropogenic Landscapes: Exploring Primate Behavioural Flexibility Across Human Contexts (pp. 247-266). Cham: Springer International Publishing.	n/a	Primates
8	Chapter	Beck, B.B.; Rapaport, L.G.; Price, M.R.S.; Wilson, A.C. Reintroduction of Captive-Born Animals. In Creative Conservation; Olney, P.J.S., Mace, G.M., Feistner, A.T.C., Eds.; Springer Netherlands: Dordrecht, 1994; pp. 265–286 ISBN 978-94-010-4311-3.	n/a	Animals (generally)
9	Chapter	Cui, Q. M., & Xu, H. G. (2021). The valuation of ethical encounters with elephants. In The elephant tourism business (pp. 111-122). Wallingford UK: CABI.	n/a	Elephants
10	Chapter	Donaldson, S. (2024). Sanctuary Communities. In: Athanassakis, Y., Larue, R., O'Donohue, W. (eds) The Plant-based and Vegan Handbook. Springer, Cham. https://doi.org/10.1007/978-3-031-63083-5_3	n/a	Animals (generally)
11	Chapter	Emmerman, K. (2014). Sanctuary, not remedy. in The Ethics of Captivity. Gruen, L. (Ed.). Oxford University Press on Demand. 213-230.	n/a	Primates (Chimpanzees)
12	Chapter	Green, R. J. (2020). 6 The Future of Captive Wildlife: Useful and Enjoyable for Animals and Visitors?. Wildlife Tourism Futures: Encounters with Wild, Captive and Artificial Animals, 4.	n/a	Wild Animals (generally)
13	Chapter	Higginbottom, K. (2004). Wildlife tourism: An introduction. Wildlife tourism: Impacts, management and planning, 1-14.	n/a	Wild Animals (generally)

14	Chapter	Irvine, L. (2017). Animal sheltering. The Oxford handbook of animal studies, 98-112.	n/a	Animals (generally)
15	Chapter	Jones, M. (2014). Captivity in the context of a sanctuary for formerly farmed animals. The ethics of captivity, 90-101.	n/a	Domesticated Animals
16	Chapter	Pabel, A., & Mkono, M. (2021). Moral tensions for elephant visitors. In The elephant tourism business (pp. 99-110). Wallingford UK: CABI.	n/a	Elephants
17	Chapter	Pachirat, T. (2018). Sanctuary. In Critical Terms for Animal Studies. Gruen, L. (Ed.). University of Chicago Press.	n/a	Animals (generally)
18	Chapter	Rizzolo, J. B. (2020). The Rise of selfie safaris and the future (s) of wildlife tourism. Wildlife tourism futures. Channel View Publications.	n/a	Wild Animals (generally)
19	Chapter	Rosenfeld, H. (2022). Sanctuaries as multispecies safe spaces. In Feminist Animal Studies (pp. 165-182). Routledge.	n/a	Domesticated Birds
20	Chapter	Ross, S. R. (2014). Captive chimpanzees. in The Ethics of Captivity. Gruen, L. (Ed.). Oxford University Press on Demand. 57-76.	n/a	Primates (Chimpanzees)
21	Chapter	Russon, A. E., Smith, J. J., & Adams, L. (2016). Managing human–orangutan relationships in rehabilitation. Ethnoprimatology: Primate conservation in the 21st century, 233-258.	n/a	Primates (Orangutans)
22	Chapter	Sadashige, J. (2024). 5 Hands Off the Herd: Negotiating Tourist Desires and Animal Welfare at Elephant Sanctuary. Emerging Voices for Animals in Tourism, 46.	n/a	Elephants
23	Chapter	Schmidt-Burbach, J. (2018). Elephants 11 and Tourism. Tourism and animal welfare, 112.	n/a	Elephants
24	Chapter	Schuurman, N. (2024). 4 Volunteer Tourism and Dog Rehoming. Emerging Voices for Animals in Tourism, 34.	n/a	Dogs
25	Chapter	Speiran, S.I. 11 Monkey See, Monkey Do: The Work of Primates in Costa Rican Sanctuaries. In Exploring non-human work in tourism; Kline, C., Rickly, J.M., Eds.; De Gruyter, 2021; pp. 181–206 ISBN 978-3-11-066405-8.	n/a	Primates
26	Chapter	Speiran, S.I.; Jeyaraj-Powell, T.; Kauffman, L.; Rodrigues, M.A. Rescue, Rehabilitation, and Reintroduction. In Primates in An-thropogenic Landscapes: Exploring Primate Behavioural Flexibility Across Human Contexts; McKinney, T., Waters, S., Rodrigues, M.A., Eds.; Springer International Publishing: Cham, 2023; pp. 267–287 ISBN 978-3-031-11736-7.	n/a	Primates
27	Chapter	Thies, T. Q. (2010). History and Function of US Sanctuaries. In Tigers of the World (pp. 215-221). William Andrew Publishing.	n/a	Tigers
28	Chapter	Thomsen, B. (2021). Wolf ecotourism: A posthumanist approach to wildlife ecotourism. In Routledge handbook of ecotourism (pp. 117-131). Routledge.	n/a	Wolves
29	Magazine Article	Marino, L., Bradshaw, G., & Malamud, R. (2009). The captivity industry. Best Friends, 25, 25-27.	n/a	Animals (generally)

30	IUCN Publication	Dempsey, A., & McKinney, T. Watching Captive Primates in Zoos and Sanctuaries. Responsible Primate-Watching for Tourists, 36. From the IUCN SSC Primate Specialist Group Section on Human-Primate Interactions	n/a	Primates
		**Peer-Reviewed Journals**		
	**Journal Title**	**Citation**	**Keywords**	**Animal Studied**
26	*Acta Commercii*	Viljoen, A. H., & Kruger, M. (2020). Could conservation management be prioritised during captive wildlife experiences?. Acta Commercii, 20(1), 1-14.	captive wildlife experiences; conservation management; sanctuaries; rehabilitation centres; visitor experience; interpretation needs; visitor motive	Wild Animals (generally)
27	*American Journal of Physical Anthropology*	Muehlenbein M. P. (2017). Primates on display: Potential disease consequences beyond bushmeat. American journal of physical anthropology, 162 Suppl 63, 32–43. https://doi.org/10.1002/ajpa.23145	Monkeyland Primate Sanctuary; Saint Kitts; Sepilok Orangutan Rehabilitation Centre; Takasakiyama Monkey Park; anthroponoses; biophilia; bushmeat; companion animals; ecotourism; emerging infectious diseases; environmental attitudes; haptics; pets; primates; zoonoses; zoos	Primates
28	*American Journal of Primatology*	Morimura, N., Idani, G., & Matsuzawa, T. (2011). The first chimpanzee sanctuary in Japan: an attempt to care for the "surplus" of biomedical research. American journal of primatology, 73(3), 226–232. https://doi.org/10.1002/ajp.20887	n/a	Primates (Chimpanzees)
29	*American Journal of Primatology*	Neufuss, J., Humle, T., Cremaschi, A., & Kivell, T. L. (2017). Nut-cracking behaviour in wild-born, rehabilitated bonobos (Pan paniscus): a comprehensive study of hand-preference, hand grips and efficiency. American journal of primatology, 79(2), 1–16. https://doi.org/10.1002/ajp.22589	hand grips; laterality; manual dexterity; nut-cracking; tool-use	Primates
30	*American Journal of Primatology*	Farmer K. H. (2002). Pan-African sanctuary alliance: status and range of activities for great ape conservation. American journal of primatology, 58(3), 117–132. https://doi.org/10.1002/ajp.10054	n/a	Primates
31	*American Journal of Primatology*	Rosati, A. G., Sabbi, K. H., Bryer, M. A. H., Barnes, P., Rukundo, J., Mukungu,… Machanda, Z. P. (2023). Observational approaches to chimpanzee behavior in an African sanctuary: Implications for research, welfare, and capacity-building. American journal of primatology, 85(9), e23534. https://doi.org/10.1002/ajp.23534	behavior; capacity-building; observations; sanctuaries; welfare	Primates
32	*American Journal of Primatology*	McEwen, E. S., Warren, E., Tenpas, S., Jones, B., Durdevic, K., Rapport Munro, E., & Call, J. (2022). Primate cognition in zoos: Reviewing the impact of zoo-based research over 15 years. American journal of primatology, 84(10), e23369. https://doi.org/10.1002/ajp.23369	methodology; primate cognition; zoo research	Primates
33	*American Journal of Primatology*	Trayford, H.R.; Farmer, K.H. Putting the Spotlight on Internally Displaced Animals (IDAs): A Survey of Primate Sanctuaries in Africa, Asia, and the Americas: A Survey of Primate Sanctuaries. Am. J. Primatol. 2013, 75, 116–134, doi:10.1002/ajp.22090.	n/a	Primates
34	*American Journal of Primatology*	Ross, S. R., Hansen, B. K., Hopper, L. M., & Fultz, A. (2019). A unique zoo-sanctuary collaboration for chimpanzees. American journal of primatology, 81(5), e22941. https://doi.org/10.1002/ajp.22941	n/a	Primates (Chimpanzees)
35	*American Quarterly*	Hua, J.; Ahuja, N. Chimpanzee Sanctuary: “Surplus” Life and the Politics of Transspecies Care. American Quarterly 2013, 65, 619–637, doi:10.1353/aq.2013.0043.	n/a	Primates (Chimpanzees)
36	*Animal Studies Journal*	Doyle, C. (2017). Captive wildlife sanctuaries: definition, ethical considerations and public perception. Animal Studies Journal, 6(2), 55-85.	n/a	Wild Animals (generally)
37	*Animal Studies Journal*	Abrell, E. (2017). Introduction: Interrogating captive freedom: The possibilities and limits of animal sanctuaries. Animal Studies Journal, 6(2), 1-8.	n/a	Animals (generally)
38	*Animal Studies Journal*	Winders, D. (2017). Captive wildlife at a crossroads–sanctuaries, accreditation, and humane-washing. Animal Studies Journal, 6(2).	n/a	Wild Animals (generally)
39	*Animal Studies Journal*	Fleury, E. (2017). Money for monkeys, and more: Ensuring sanctuary retirement of nonhuman primates. Animal Studies Journal, 6(2), 30-54.	Primate sanctuary, sanctuary retirement, primate retirement, monkey, ape, nonhuman primate, primate research, primate entertainer, primate pet	Primates

40	*Animal Studies Journal*	Fusari, S. (2017). What is an animal sanctuary? Evidence from applied linguistics. Animal Studies Journal, 6(2), 137-160.	Applied linguistics, corpus linguistics, critical discourse analysis, dictionaries, Human-Animal Studies	Animals (generally)
41	*Animal Studies Journal*	Fultz, A. (2017). A guide for modern sanctuaries with examples from a captive chimpanzee sanctuary. Animal Studies Journal, 6(2), 9-29.	chimpanzee, welfare, sanctuary, ethics, wellbeing	Primates (Chimpanzees)
42	*Animals*	Flower, E. K., Burns, G. L., & Jones, D. N. (2021). How tourist preference and satisfaction can contribute to improved welfare standards at elephant tourism venues in Thailand. Animals, 11(4), 1094.	Tourist satisfaction; tourist preference; animal welfare; Asian elephant	Elephants
43	*Animals*	Sarchese, V.; Di Profio, F.; Palombieri, A.; Friedrich, K.G.; Robetto, S.; Banyai, K.; Marsilio, F.; Martella, V.; Di Martino, B. Circoviridae Survey in Captive Non-Human Primates, Italy. Animals 2024, 14, 881. https://doi.org/10.3390/ani14060881	Circoviridae; cyclovirus; non-human primates	Primates
44	*Animals*	Chaiyarat, R., Sriphonkrang, N., Khamsirinan, P., Nakbun, S., & Youngpoy, N. (2023). Age structure, development and population Viability of Banteng (Bos javanicus) in captive breeding for ex-situ conservation and reintroduction. Animals, 13(2), 198.	banteng; captive breeding; population viability analysis; reintroduction program; Salakphra Wildlife Sanctuary	Bovines (Bantengs)
45	*Animals*	Bruck, J.N. The Cetacean Sanctuary: A Sea of Unknowns. Animals 2024, 14, 335. https://doi.org/10.3390/ani14020335	cetacean; sanctuary; whale; welfare; sea pen; naturalistic enclosures	Cetaceans
46	*Animals*	Jensvold, M.L.; Dombrausky, K.; Collins, E. Sign Language Studies with Chimpanzees in Sanctuary. Animals 2023, 13, 3486. https://doi.org/10.3390/ani13223486	chimpanzee; sign language; sanctuary; ASL; vocabulary; communicative function	Primates (Chimpanzees)
47	*Animals*	Fultz, A.; Lewis, R.; Kelly, L.; Garbarino, J. Behavioral Welfare Research for the Management of Sanctuary Chimpanzees (Pan troglodytes). Animals 2023, 13, 2595. https://doi.org/10.3390/ani13162595	animal welfare; behavior; research; observations; behavioral management	Primates (Chimpanzees)
48	*Animals*	Brando, S.; Norman, M. Handling and Training of Wild Animals: Evidence and Ethics-Based Approaches and Best Practices in the Modern Zoo. Animals 2023, 13, 2247. https://doi.org/10.3390/ani13142247	animal wellbeing; animal training; zoo animals; learning; positive reinforcement; human–animal relationship; capacity building; refinement; 3Rs	Wild Animals (generally)
49	*Animals*	Bennamoun, N.; Campera, M.; Tully, G.; Nekaris, K.A.I. COVID-19's Impact on the Pan African Sanctuary Alliance: Challenging Times and Resilience from Its Members. Animals 2023, 13, 1486. https://doi.org/10.3390/ani13091486	SARS-CoV-2; conservation; sanctuary; primates; management; ecotourism; best practices; pandemic	Primates
50	*Animals*	Ross, S. R., Joshi, P. B., Terio, K. A., & Gamble, K. C. (2022). A 25-Year Retrospective Review of Mortality in Chimpanzees (Pan troglodytes) in Accredited U.S. Zoos from a Management and Welfare Perspective. Animals : an open access journal from MDPI, 12(15), 1878. https://doi.org/10.3390/ani12151878	Pan troglodytes; aggression; chimpanzee; death; mortality; necropsy; retrospective; welfare; zoos	Primates (Chimpanzees)
51	*Animals*	Fultz, A.; Yanagi, A.; Breaux, S.; Beaupre, L.; Naitove, N. How Sanctuary Chimpanzees (Pan troglodytes) Use Space after Being Introduced to a Large Outdoor Habitat. Animals 2023, 13, 961. https://doi.org/10.3390/ani13060961	space use; indoor/outdoor enclosures; species-typical behavior; welfare	Primates (Chimpanzees)
52	*Animals*	Ayuso, P.R.; Feliu, O.; Riba, D.; Crailsheim, D. Listening to Their Nights: Sleep Disruptions in Captive Housed Chimpanzees Affect Their Daytime Behavior. Animals 2023, 13, 696. https://doi.org/10.3390/ani13040696	chimpanzee; nocturnal activity; sleep disruption; sound recording; vocalization; sanctuary; welfare; agonistic behavior; temperature; humidity	Primates (Chimpanzees)
53	*Animals*	Pascual A, Kalcher-Sommersguter E, Riba D, Crailsheim D. Long-Term Assessment of Captive Chimpanzees: Influence of Social Group Composition, Seasonality and Biographic Background. Animals. 2023; 13(3):424. https://doi.org/10.3390/ani13030424	chimpanzee; social network analysis; behavior; group alterations; captive care; group composition; seasonality; adverse early life experiences	Primates (Chimpanzees)
54	*Animals*	Clay, A.W.; Ross, S.R.; Lambeth, S.; Vazquez, M.; Breaux, S.; Pietsch, R.; Fultz, A.; Lammey, M.; Jacobson, S.L.; Perlman, J.E.; et al. Chimpanzees (Pan troglodytes) in U.S. Zoos, Sanctuaries, and Research Facilities: A Survey-Based Comparison of Species-Typical Behaviors. Animals 2023, 13, 251. https://doi.org/10.3390/ani13020251	primate; chimpanzee; welfare; species-typical behavior; ape	Primates (Chimpanzees)
55	*Animals*	Lopresti-Goodman, S.M.; Villatoro-Sorto, B. The Benefits and Challenges of Conducting Primate Research in Different Settings. Animals 2023, 13, 133. https://doi.org/10.3390/ani13010133	primates; research ethics; welfare; cognition; behavior; laboratories; zoos; sanctuaries; field studies	Primates
56	*Animals*	Von Essen, E., Lindsjö, J., & Berg, C. (2020). Instagranimal: Animal welfare and animal ethics challenges of animal-based tourism. Animals, 10(10), 1830.	animal welfare; tourism; ethics; guidelines; cultural relativism; compassionate; 3Rs	Animals (generally)

57	*Animals*	López-Álvarez, J., Sanjorge, Y., Soloaga, S., Crailsheim, D., & Llorente, M. (2019). Looking for Visitor's Effect in Sanctuaries: Implications of Guided Visitor Groups on the Behavior of the Chimpanzees at Fundació Mona. Animals : an open access journal from MDPI, 9(6), 347. https://doi.org/10.3390/ani9060347	behavior; captivity; chimpanzee; human interaction; sanctuary; visitor effect; welfare	Primates (Chimpanzees)
58	*Animals*	van Leeuwen, E.J.C.; Bruinstroop, B.M.C.; Haun, D.B.M. Early Trauma Leaves No Social Signature in Sanctuary-Housed Chimpanzees (Pan troglodytes). Animals 2023, 13, 49. https://doi.org/10.3390/ani13010049	chimpanzees; welfare; social deprivation; trauma; coping; sanctuary	Primates (Chimpanzees)
59	*Animals*	Jensvold, M.L. A Preliminary Assessment of Compassion Fatigue in Chimpanzee Caregivers. Animals 2022, 12, 3506. https://doi.org/10.3390/ani12243506	ProQOL; compassion fatigue; chimpanzee caregiver; husbandry; compassion satisfaction; secondary traumatic stress; burnout; animal worker	Primates (Chimpanzees)
60	*Animals*	Chorney, S.; DeFalco, A.; Jacquet, J.; LaFrance, C.; Lary, M.; Pirker, H.; Franks, B. Poor Welfare Indicators and Husbandry Practices at Lion (Panthera Leo) “Cub-Petting” Facilities: Evidence from Public YouTube Videos. Animals 2022, 12, 2767. https://doi.org/10.3390/ani12202767	lion protection; compassionate conservation; wildlife tourism; animal welfare; human–animal interactions; social media; content analysis; species-specific behavior; wildlife exploitation	Lions
61	*Animals*	Fultz, A.; Yanagi, A.; Breaux, S.; Beaupre, L. Aggressive, Submissive, and Affiliative Behavior in Sanctuary Chimpanzees (Pan Troglodytes) During Social Integration. Animals 2022, 12, 2421. https://doi.org/10.3390/ani12182421	chimpanzee; introduction; social integration; socialization; behavior	Primates (Chimpanzees)
62	*Animals*	Lopresti-Goodman, S. M., & Villatoro-Sorto, B. (2022). The Benefits and Challenges of Conducting Primate Research in Different Settings. *Animals*, 13(1), 133. https://doi.org/10.3390/ani13010133	behavior; cognition; field studies; laboratories; primates; research ethics; sanctuaries; welfare; zoos.	Primates
63	*Animals*	Greeson, J.L.; Gabriel, K.I.; Mulcahy, J.B.; King Hendrickson, B.; Lonborg, S.D.; Holloway, J.C. An Evaluation of Ethograms Measuring Distinct Features of Enrichment Use by Captive Chimpanzees (Pan troglodytes). Animals 2022, 12, 2029. https://doi.org/10.3390/ani12162029	primate behavior; environmental enrichment; Pan troglodytes; social contexts; object manipulation; individual preferences; principal component analysis; chimpanzee; primate welfare	Primates (Chimpanzees)
64	*Animals*	Feliu, O.; Masip, M.; Maté, C.; Sánchez-López, S.; Crailsheim, D.; Kalcher-Sommersguter, E. Behavioural Development of Three Former Pet Chimpanzees a Decade after Arrival at the MONA Sanctuary. Animals 2022, 12, 138. https://doi.org/10.3390/ani12020138	chimpanzee; Pan troglodytes; activity budget; sanctuary; re-socialization; well-being; early life experience; pet and entertainment	Primates (Chimpanzees)
65	*Animals*	Kelemen, Z.; Grimm, H.; Long, M.; Auer, U.; Jenner, F. Recumbency as an Equine Welfare Indicator in Geriatric Horses and Horses with Chronic Orthopaedic Disease. Animals 2021, 11, 3189. https://doi.org/10.3390/ani11113189	welfare; horse; equine; sleep; lying; time budget; locomotion; geriatric; orthopedic; recumbency	Horses
66	*Animals*	Padrell, M.; Amici, F.; Córdoba, M.P.; Giberga, A.; Broekman, A.; Almagro, S.; Llorente, M. Artificial Termite-Fishing Tasks as Enrichment for Sanctuary-Housed Chimpanzees: Behavioral Effects and Impact on Welfare. Animals 2021, 11, 2941. https://doi.org/10.3390/ani11102941	chimpanzees; behavior; enrichment; tool use; welfare	Primates (Chimpanzees)
67	*Animals*	Charalambous, R.; Simonato, T.; Peel, M.; Narayan, E.J. Physiological Stress in Rescued Wild Koalas (Phascolarctos cinereus) Being Held in a Rehabilitation Sanctuary: A Pilot Study. Animals 2021, 11, 2864. https://doi.org/10.3390/ani11102864	faeces; fur; glucocorticoids; stress	Koalas
68	*Animals*	Anderson, N.; Amarasekaran, B.; Riba, D. An Investigation into the Influence of Different Types of Nesting Materials upon the Welfare of Captive Chimpanzees (Pan troglodytes). Animals 2021, 11, 1835. https://doi.org/10.3390/ani11061835	bedding; nesting materials; environmental enrichment; welfare; captive chimpanzees	Primates (Chimpanzees)
69	*Animals*	Burton, E.; Tribe, A. The Rescue and Rehabilitation of Koalas (Phascolarctos cinereus) in Southeast Queensland. Animals 2016, 6, 56. https://doi.org/10.3390/ani6090056	koalas; rehabilitation; release	Koalas
70	*Animals*	Costa, M.M.; Pinto da Cunha, N.; Hagnauer, I.; Venegas, M. A Retrospective Analysis of Admission Trends and Outcomes in a Wildlife Rescue and Rehabilitation Center in Costa Rica. Animals 2024, 14, 51. https://doi.org/10.3390/ani14010051	wildlife; release; mortality	Wild Animals (generally)
71	*Animals*	Ongman, L.; Colin, C.; Raballand, E.; Humle, T. The “Super Chimpanzee”: The Ecological Dimensions of Rehabilitation of Orphan Chimpanzees in Guinea, West Africa. Animals 2013, 3, 109-126. https://doi.org/10.3390/ani3010109	chimpanzee; rehabilitation; socially-biased learning; abnormal behaviors; reintroduction; sanctuary; bush-outings	Primates (Chimpanzees)
72	*Annals of the Association of American Geographers*	Collard, R. C. (2014). Putting animals back together, taking commodities apart. Annals of the Association of American Geographers, 104(1), 151-165.	commodification; decommodification; global live wildlife trade; human–animal relations; wildlife rehabilitation	Wild Animals (generally)

73	*Anthrozoös*	Nunes, V. F., Lopes, P. F. M., & Ferreira, R. G. (2023). #capuchinmonkeys on Social Media: A Threat for Species Conservation. Anthrozoös, 36(4), 665–683. https://doi.org/10.1080/08927936.2023.2210440	Ethnoprimatology; humananimal interaction; pet trade; social media; wildlife trafficking	Primates (Capuchins)
74	*Anthrozoös*	James, A., & Pieterse, J. (2022). Seeking Sanctuary: Creating a New Utopia on a Donkey Farm. Anthrozoös, 35(2), 273-291.	Cosmology, donkeys, human-animal interaction, morality, social order, veganism	Donkeys
75	*Anthrozoös*	Woods, B. (2002). Good zoo/bad zoo: Visitor experiences in captive settings. Anthrozoös, 15(4), 343-360.	captive wildlife; visitor experiences; wildlife tourism	Wild Animals (generally)
76	*Applied Animal Behaviour Science*	Robinson, L. M., Crudge, B., Lim, T., Roth, V., Gartner, M., Naden, K., ... & Descovich, K. (2022). Limitations and challenges of adapting subjective keeper questionnaires to non-Western sanctuary settings. Applied Animal Behaviour Science, 251, 105627.	Personality; Rehabilitation; Behaviour; Rescue; Temperament; Anthrozoology; Captive	Bears
77	*Applied Animal Behaviour Science*	Stagni, E.; Brscic, M.; Contiero, B.; Kirchner, M.; Sequeira, S.; Hartmann, S. Development of a Fixed List of Terms for Qualitative Behavioural Assessment of Brown Bear (Ursus Arctos) in Sanctuaries. Applied Animal Behaviour Science 2022, 246, 105523, doi:10.1016/j.applanim.2021.105523.	Animal welfare; Brown bear; Qualitative behavioural assessment; Sanctuary	Bears
78	*Applied Animal Behaviour Science*	Cronin, K. A., West, V., & Ross, S. R. (2016). Investigating the relationship between welfare and rearing young in captive chimpanzees (Pan troglodytes). Applied Animal Behaviour Science, 181, 166-172.	n/a	Primates (Chimpanzees)
79	*Asia Pacific Viewpoint*	Cui, Q., & Xu, H. (2019). Situating animal ethics in Thai elephant tourism. Asia Pacific Viewpoint, 60(3), 267-279.	n/a	Elephants
80	*Asia-Pacific Journal of Innovation in Hospitality and Tourism*	Rathakrishnan, T., Ramachandran, S., Ling, S. M., Shuib, A., Afandi, S. H. M., Kunasekaran, P., & Prabhakaran, S. (2020). Wildlife memorable tourism experiences as antecedents of visitor loyalty at Sepilok Orangutan Rehabilitation Centre (SORC), Sabah, Malaysia. Asia-Pacific Journal of Innovation in Hospitality and Tourism (APJIHT), 9(1), 47-71.	Wildlife tourism, conservation, tourist intention, experience economy, endangered species, tourism marketing	Primates (Orangutans)
81	*Behavioural Sciences*	Lopresti-Goodman, S. M., Kameka, M., & Dube, A. (2012). Stereotypical behaviors in chimpanzees rescued from the african bushmeat and pet trade. Behavioral sciences (Basel, Switzerland), 3(1), 1–20. https://doi.org/10.3390/bs3010001	abnormal behaviors; bushmeat; chimpanzees; post-traumatic stress disorder; stereotypical behaviors	Primates (Chimpanzees)
82	*Biodiversitas Journal of Biological Diversity*	Siregar, P. G., & Setia, T. M. (2019). Implementation of animal welfare in tiger sanctuary, Barumun Nagari Wildlife Sanctuary, North Sumatra, Indonesia. Biodiversitas Journal of Biological Diversity, 20(10).	n/a	Tigers
83	*Biodiversity & Conservation*	Ferrie, G. M., Farmer, K. H., Kuhar, C. W., Grand, A. P., Sherman, J., & Bettinger, T. L. (2014). The social, economic, and environmental contributions of Pan African Sanctuary Alliance primate sanctuaries in Africa. Biodiversity and conservation, 23, 187-201.	n/a	Primates
84	*Biological Conservation*	Miller, T. K., Pierce, K., Clark Jr, E. E., & Primack, R. B. (2023). Wildlife rehabilitation records reveal impacts of anthropogenic activities on wildlife health. Biological Conservation, 286, 110295.	Toxicants; Endangered species; Climate change; Extreme weather events; Lead; Pesticides	Wild Animals (generally)
85	*Biological Conservation*	Palmer, A. (2018). Kill, incarcerate, or liberate? Ethics and alternatives to orangutan rehabilitation. Biological Conservation, 227, 181-188.	Rehabilitation; Reintroduction; Apes; Orangutans; Ethics; Compassionate conservation	Primates (Orangutans)
86	*Biology Letters*	Ross, S. R., & Leinwand, J. G. (2020). A review of research in primate sanctuaries. Biology letters, 16(4), 20200033.	n/a	Primates
87	*Biotropica*	Chaves, A., Montecino-Latorre, D., Alcázar, P., & Suzán, G. (2021). Wildlife rehabilitation centers as a potential source of transmission of SARS-CoV-2 into native wildlife of Latin America. Biotropica, 53(4), 987-993.	n/a	Wild Animals (generally)
88	*Conservation Biology*	Harrington, L. A., Moehrenschlager, A., Gelling, M., Atkinson, R. P., Hughes, J., & Macdonald, D. W. (2013). Conflicting and complementary ethics of animal welfare considerations in reintroductions. Conservation Biology, 27(3), 486-500.	n/a	Wild Animals (generally)
89	*Current Issues in Tourism*	Fennell, D. A., Kline, C., Mkono, M., Grimwood, B. S. R., Sheppard, V. A., Dashper, K.,… Madrid, R. (2024). Tourism, animals & the vacant niche: a scoping review and pedagogical agenda. Current Issues in Tourism, 27(22), 3820–3848. https://doi.org/10.1080/13683500.2023.2280704	Tourism; Animal ethics; Pedagogy; Vacant niche; Expanding consciousness	Animals (generally)

90	*Current Issues in Tourism*	Kontogeorgopoulos, N. Wildlife Tourism in Semi-Captive Settings: A Case Study of Elephant Camps in Northern Thailand. Current Issues in Tourism 2009, 12, 429–449, doi:10.1080/13683500903042873.	wildlife tourism; Thailand; elephants; animal rights	Elephants
91	*Elephants Under Human Care*	Rees, P. A. (2021). The future of elephants in captivity. Elephants Under Human Care, 313.	Elephant ranching; rewilding; elephant sanctuaries; elephant welfare; elephants in zoos; elephant genetics; elephants and climate change; release of elephants to wild	Elephants
92	*Environment and Planning E: Nature and Space*	Rosenfeld, H. (2024). Deviant accumulation at farmed animal sanctuaries. Environment and Planning E: Nature and Space, 7(1), 271-287. https://doi-org.proxy.queensu.ca/10.1177/25148486231167870	n/a	Domesticated Animals
93	*Folia Primatologica*	Fultz, A., Brent, L., Breaux, S. D., & Grand, A. P. (2013). An evaluation of nest-building behavior by sanctuary chimpanzees with access to forested habitats. Folia primatologica; international journal of primatology, 84(6), 405–420. https://doi.org/10.1159/000353900	Nest-building; Sanctuary; Vegetation; Bed; Great ape; Captive; Nests	Primates (Chimpanzees)
94	*Fowler's Zoo and Wild Animal Medicine Current Therapy*	Bezner, J. (2019). Medical aspects of chimpanzee rehabilitation and sanctuary medicine. In Fowler's Zoo and Wild Animal Medicine Current Therapy, Volume 9 (pp. 574-580). WB Saunders.	n/a	Primates (Chimpanzees)
95	*Frontiers in Veterinary Science*	Thompson Iritani, S., Brando, S., & Hart, L. A. (2023). Editorial: Occupational stress and joy of animal care professionals in zoos, sanctuaries, farms, shelters, and laboratory animal facilities. Frontiers in veterinary science, 10, 1164483. https://doi.org/10.3389/fvets.2023.1164483	animal welfare; burnout; caregiving; compassion fatigue; empathic strain; mental health	Animals (generally)
96	*Human-Animal Interactions*	Compitus,Katherine and Bierbower,Sonya M., hai.2024.0016, Human-Animal Interactions, doi:10.1079/hai.2024.0016, CABI, Cow cuddling: Cognitive considerations in bovine-assisted therapy, (2024)	n/a	Cows
97	*Human-Animal Interactions*	Nadal,Zaida and Ferrari,Mónica and Lora,Julián and Revollo,Alicia and Nicolas,Florencia and Astegiano,Santiago and Díaz Videla,Marcos, hai.2022.0003, Human-animal interaction bulletin, doi:10.1079/hai.2022.0003, CABI International, Noah's Syndrome: Systematic Review of Animal Hoarding Disorder, (2022)	n/a	Domestic Cats
98	*Human-Animal Interactions*	Rocha,Sílvia and Gaspar,Augusta and Esteves,Francisco, hai.2016.0007, Human-animal interaction bulletin, doi:10.1079/hai.2016.0007, CABI International, Developing Children's Ability to Recognize Animal Emotions – What Does It Take? A Study at the Zoo, (2016)	n/a	Animals (generally)
99	*Human–Wildlife Interactions*	Smith, W. E., Pekins, P. J., Timmins, A. A., & Kilham, B. (2016). Short-term fate of rehabilitated orphan black bears released in New Hampshire. Human-Wildlife Interactions, 10(2), 258-267.	black bear, orphaned bears, rehabilitation, release, Ursus americanus	Bears
100	*Human–Wildlife Interactions*	Perry, D. J., & Averka, J. P. (2020). Caring for the circle of life: Wildlife rehabilitation and sanctuary care. Human–Wildlife Interactions, 14(2), 18.	caregivers, conservation, human–wildlife coexistence, Maine, Massachusetts, New Hampshire, wildlife, wildlife rehabilitation, wildlife sanctuary	Wild Animals (generally)
101	*International Journal Avian Wildlife Biology*	Bais, B., Tak, L., & Mahla, S. (2017). Study of preventive health measures for wildlife in captivity: a review of management approaches. Int J Avian Wildl Biol, 2(3), 73-75.	wildlife, captivity, animals, management	Wild Animals (generally)
102	*International Journal for Research in Applied Science & Engineering Technology*	Sharma, K., Sharma, A., Sharma, S., Prakash, B., Jacob, S., & Hasani, F. Health Issues in Animals in Zoo as Compared to Wildlife Sanctuary. International Journal for Research in Applied Science & Engineering Technology (IJRASET),Volume 10 Issue V May 2022	Behaviors, Captivity, infection, intellectual, Zoochoisis.	Wild Animals (generally)
103	*International Journal of Primatology*	Palmer, A., & Malone, N. (2018). Extending Ethnoprimatology: Human-Alloprimate Relationships in Managed Settings. International journal of primatology, 39(5), 831–851. https://doi.org/10.1007/s10764-017-0006-6	Conservation; Ethnoprimatology; Human–primate interfaces; Rehabilitation and reintroduction; Sanctuaries; Zoos	Primates
104	*Journal for Nature Conservation*	Sherman, J., Ancrenaz, M., & Meijaard, E. (2020). Shifting apes: Conservation and welfare outcomes of Bornean orangutan rescue and release in Kalimantan, Indonesia. Journal for Nature Conservation, 55, 125807.	Bornean orangutan; Conservation; Reintroduction; Translocation; Law enforcement	Primates (Orangutans)
105	*Journal of Applied Animal Welfare Science*	Laule, G., & Whittaker, M. (2007). Enhancing nonhuman primate care and welfare through the use of positive reinforcement training. Journal of applied animal welfare science : JAAWS, 10(1), 31–38. https://doi.org/10.1080/10888700701277311	n/a	Primates
106	*Journal of Applied Animal Welfare Science*	Pierce, J., & Bekoff, M. (2018). A postzoo future: Why welfare fails animals in zoos. Journal of Applied Animal Welfare Science, 21(sup1), 43-48.	Zoos; freedom; captivity; reform; ethics	Wild Animals (generally)

107	*Journal of Applied Animal Welfare Science*	Brent L. (2007). Life-long well being: applying animal welfare science to nonhuman primates in sanctuaries. Journal of applied animal welfare science : JAAWS, 10(1), 55–61. https://doi.org/10.1080/10888700701277626	n/a	Primates
108	*Journal of Applied Animal Welfare Science*	Noon C. (1999). Chimpanzees and retirement. Journal of applied animal welfare science : JAAWS, 2(2), 141–146. https://doi.org/10.1207/s15327604jaws0202_6	n/a	Primates (Chimpanzees)
109	*Journal of Ecotourism*	Tomassini, L.; Bertella, G.; Grasso, C.; Lenzi, C. The Space of Animal Justice in Wildlife Sanctuaries: A Posthuman Perspective. Journal of Ecotourism 2022, 1–18, doi:10.1080/14724049.2022.2122480.	Animal space; animal justice; posthumanism; wildlife sanctuaries; eco-tourism	Wild Animals (generally)
110	*Journal of Ecotourism*	Taylor, M., Hurst, C. E., Stinson, M. J., & Grimwood, B. S. (2020). Becoming care-full: Contextualizing moral development among captive elephant volunteer tourists to Thailand. Journal of Ecotourism, 19(2), 113-131.	Animal space; animal justice; posthumanism; wildlife sanctuaries; eco-tourism	Elephants
111	*Journal of Ecotourism*	Bendell, B. L. (2024). Tourist crossing: examining the intersection of wildlife tourism attractions & animal welfare in Costa Rica. Journal of Ecotourism, 1-11.	Animal welfare; ethical selfies; rescues and sanctuaries; coalition building; government support; wildlife tourism attraction	Wild Animals (generally)
112	*Journal of Ecotourism*	Thomsen, B., Copeland, K., Fennell, S. R., Thomsen, J., Harte, M., Deshwalt, A., ... & Muurlink, O. (2023). The promise of posthumanism in wildlife ecotourism: A set of case studies of veterinarians' role at wildlife rehabilitation centers in Costa Rica. Journal of Ecotourism, 1-19.	Posthumanism; wildlife ecotourism; multispecies livelihoods; veterinarian medicine; wildlife rehabilitation	Wild Animals (generally)
113	*Journal of Land Use & Environmental Law*	Landwerlen, A. (2023). Deposing a Tiger King: How the Big Cat Public Safety Act is Changing the Legal Framework of Private Pet Ownership & Commercial Exhibition of Exotic Species. J. Land Use & Env't L., 39, 233.	n/a	Tigers & 'Big Cats'
114	*Journal of Sustainable Tourism*	Thomsen, B., Thomsen, J., Copeland, K., Coose, S., Arnold, E., Bryan, H., ... & Chalich, G. (2023). Multispecies livelihoods: A posthumanist approach to wildlife ecotourism that promotes animal ethics. Journal of Sustainable Tourism, 31(5), 1195-1213.	Wildlife ecotourism; multispecies livelihoods; sustainable development; biodiversity conservation; posthumanism; wildlife-human coexistence	Wild Animals (generally)
115	*Journal of Sustainable Tourism*	Rizzolo, J. B. (2023). Wildlife tourism and consumption. Journal of Sustainable Tourism, 31(5), 1181-1194.	Wildlife; consumption; selfie; safari; elephant ride; food; souvenir	Wild Animals (generally)
116	*Journal of Tourism Studies*	Woods, B. (1998). Animals on display: Principles for interpreting captive wildlife. Journal of Tourism Studies, 9(1), 28-39.	Children's needs; Visitor information; Visitor attention; Interpretation in zoos; Captive behaviours; Animal inactivity; Effective signs	Wild Animals (generally)
117	*Journal of Trauma & Dissociation*	Lopresti-Goodman, S. M., Bezner, J., & Ritter, C. (2015). Psychological Distress in Chimpanzees Rescued From Laboratories. Journal of trauma & dissociation : the official journal of the International Society for the Study of Dissociation (ISSD), 16(4), 349–366. https://doi.org/10.1080/15299732.2014.1003673	chimpanzees; complex posttraumatic stress disorder; posttraumatic stress disorder; psychological distress; self-injurious behaviors	Primates (Chimpanzees)
118	*The Journal of Wildlife Management*	Blair, C. D., Muller, L. I., Clark, J. D., & Stiver, W. H. (2020). Survival and conflict behavior of American black bears after rehabilitation. The Journal of Wildlife Management, 84(1), 75-84.	Black bear, days post-release, orphaned bears, rehabilitation, release, survival, Ursus	Bears
119	*Krisis*	Meijer, E. Sanctuary Politics and the Borders of the Demos: A Comparison of Human and Nonhuman Animal Sanctuaries. krisis 2021, 41, 35–48, doi:10.21827/krisis.41.2.37174.	Animal Sanctuary; City of Sanctuary; Expanded Sanctuary; Interspecies Relations; Political Animal; Philosophy; Political Philosophy	Animals (generally)
120	*Midwest Studies in Philosophy*	Abbate, C. (2022). On the ill-being of animals: From factory farm to forever home. Midwest Studies in Philosophy, 46, 325-353.	Animal welfare, ill-being, exploitation, captivity, sanctuary	Domesticated Animals
121	*New York University Law Review*	Williams, S. J. (2018). Unsafe havens: Improving third-party accreditation of wildlife sanctuaries. NYUL Rev., 93, 1351.	n/a	Wild Animals (generally)
122	*PeerJ*	Che-Castaldo, J., Havercamp, K., Watanuki, K., Matsuzawa, T., Hirata, S., & Ross, S. R. (2021). Comparative survival analyses among captive chimpanzees (Pan troglodytes) in America and Japan. PeerJ, 9, e11913. https://doi.org/10.7717/peerj.11913	America; Captivity; Chimpanzee; Japan; Life history; Life table; Longevity; Mortality; Survival analyses	Primates (Chimpanzees)
123	*PeerJ*	Feliu, O., González-Zamora, A., Riba, D., Sauquet, T., Sánchez-López, S., & Maté, C. (2023). The impact of sanctuary visits on children's knowledge and attitudes toward primate welfare and conservation. PeerJ, 11, e15074. https://doi.org/10.7717/peerj.15074	Assessment; Attitudes; Chimpanzees; Conservation; Education; Knowledge; Primates; Sanctuary; School children; Wildlife trade.	Animals (generally)

124	*PloS one*	Moorhouse, T. P., Dahlsjö, C. A., Baker, S. E., D'Cruze, N. C., & Macdonald, D. W. (2015). The customer isn't always right—conservation and animal welfare implications of the increasing demand for wildlife tourism. PloS one, 10(10), e0138939.	n/a	Wild Animals (generally)
125	*PLoS one*	Wobber, V., & Hare, B. (2011). Psychological health of orphan bonobos and chimpanzees in African sanctuaries. PloS one, 6(6), e17147. https://doi.org/10.1371/journal.pone.0017147	n/a	Primates
126	*PLoS one*	Dunay, E., Rukundo, J., Atencia, R., Cole, M. F., Cantwell, A., Emery Thompson, M., Rosati, A. G., & Goldberg, T. L. (2023). Viruses in saliva from sanctuary chimpanzees (Pan troglodytes) in Republic of Congo and Uganda. PloS one, 18(6), e0288007. https://doi.org/10.1371/journal.pone.0288007	n/a	Primates (Chimpanzees)
127	*PLoS one*	Le Flohic, G., Motsch, P., DeNys, H., Childs, S., Courage, A., & King, T. (2015). Behavioural ecology and group cohesion of juvenile western lowland gorillas (Gorilla g. gorilla) during rehabilitation in the Batéké Plateaux National Park, Gabon. PloS one, 10(3), e0119609. https://doi.org/10.1371/journal.pone.0119609	n/a	Primates
128	*PLoS one*	Leeds, A., Kakule, D., Stalter, L., Mbeke, J. K., & Fawcett, K. (2024). Group structure and individual relationships of sanctuary-living Grauer's gorillas (Gorilla beringei graueri). PloS one, 19(1), e0295561. https://doi.org/10.1371/journal.pone.0295561	n/a	Primates
129	*Politics and Animals*	Blattner, C. E., Donaldson, S., & Wilcox, R. (2020). Animal agency in community. Politics and Animals, 6(0), 1-22.	farmed animals; agency; sanctuary; intentional community; multispecies ethnography; social norms; social roles	Domesticated Animals
130	*Politics and Animals*	Donaldson, S., & Kymlicka, W. (2015). Farmed animal sanctuaries: The heart of the movement?. Politics and Animals, 1(1), 50-74.	sanctuaries; citizenship; farmed animals; domesticated animals; intentional communities	Domesticated Animals
131	*Primates: Journal of Primatology*	Padrell, M., Amici, F., Córdoba, M. P., & Llorente, M. (2022). Cognitive enrichment in a social setting: assessing the use of a novel food maze in sanctuary-housed chimpanzees. Primates; journal of primatology, 63(5), 509–524. https://doi.org/10.1007/s10329-022-00996-0	Behavior; Chimpanzees; Cognitive enrichment; Tool use; Welfare	Primates
132	*Primates: Journal of Primatology*	Padrell, M.; Amici, F.; Córdoba, M.P.; Llorente, M. Cognitive Enrichment in a Social Setting: Assessing the Use of a Novel Food Maze in Sanctuary-Housed Chimpanzees. Primates 2022, 63, 509–524, doi:10.1007/s10329-022-00996-0.	n/a	Primates
133	*Primates: Journal of Primatology*	Campbell L. A. D. (2019). Fostering of a wild, injured, juvenile by a neighbouring group: implications for rehabilitation and release of Barbary macaques confiscated from illegal trade. Primates; journal of primatology, 60(4), 339–345. https://doi.org/10.1007/s10329-019-00729-w	Consolation; Illegal wildlife trade; Primate adoption; Reintroduction; Third-party affiliation; Wildlife rehabilitation	Primates
134	*Primates: Journal of Primatology*	Funkhouser, J. A., Mayhew, J. A., Mulcahy, J. B., & Sheeran, L. K. (2021). Human caregivers are integrated social partners for captive chimpanzees. Primates; journal of primatology, 62(2), 297–309. https://doi.org/10.1007/s10329-020-00867-6	Captive welfare; Human-animal relationships; Interspecific social relationships; Pan troglodytes; Philosophies of care; Social network analysis	Primates (Chimpanzees)
135	*Primates: Journal of Primatology*	Ross, S. R., Lake, B. R., Fultz, A., & Hopper, L. M. (2021). An evaluation of thermal imaging as a welfare monitoring tool for captive chimpanzees. Primates; journal of primatology, 62(6), 919–927. https://doi.org/10.1007/s10329-021-00943-5	n/a	Primates (Chimpanzees)
136	*Science*	Grimm D. (2016). ANIMAL RESEARCH. Chimpanzee sanctuaries open door to more research. Science (New York, N.Y.), 353(6298), 433–434. https://doi.org/10.1126/science.353.6298.433	n/a	Primates (Chimpanzees)
137	*Society & Animals*	Taylor, N. (2004). In it for the nonhuman animals: Animal welfare, moral certainty, and disagreements. Society & Animals, 12(4), 317-339.	n/a	Animals (generally)
138	*Society & Animals*	Cohen, E. “Buddhist Compassion” and “Animal Abuse” in Thailand’s Tiger Temple. Society & Animals 2013, 21, 266–283, doi:10.1163/15685306-12341282.	animal abuse; animal shows; ethical treatment of animals; human-animal engagement; tigers; Tiger Temple	Tigers
139	*Society & Animals*	Perry, D. J., Averka, J. P., Johnson, C., Powell, H., & Cavanaugh, A. (2022). Visitors’ feelings toward moose and coyote in a wildlife sanctuary: transcendent feelings of animal valuation scale. society & animals, 31(7), 866-887.	wildlife; feelings; moose; coyote; wildlife sanctuary; animal welfare; coexistence	Coyotes, Moose
140	*South African Journal of Wildlife Research*	Schoene, C. U. R., & Brend, S. A. (2002). Primate sanctuaries-a delicate conservation approach. South African Journal of Wildlife Research, 32(2), 109-113.	n/a	Primates

141	*Tourism Management*	Sebele, L.S. Community-Based Tourism Ventures, Benefits and Challenges: Khama Rhino Sanctuary Trust, Central District, Botswana. Tourism Management 2010, 31, 136–146, doi:10.1016/j.tourman.2009.01.005.	Community-based tourism; Community-Based Natural Resource Management; Community participation; Khama Rhino Sanctuary Trust; Botswana	Rhinoceroses
142	*Tourism Recreation Research*	Blaer, M. (2024). Animal rescue tourism: digital technology-enhanced approaches to support voluntourist engagement, animal welfare and rights. Tourism Recreation Research, 49(3), 471-485.	Animal rescue tourism; volunteer tourism; social media; animal rights; animal welfare	Animals (generally)
143	*Tourism Recreation Research*	Broad, S. (2003). Living the Thai life—a case study of volunteer tourism at the Gibbon Rehabilitation Project, Thailand. Tourism recreation research, 28(3), 63-72.	volunteer tourism; Thai culture; Gibbon Rehabilitation Project; volunteer experience	Primates (Gibbons)
144	*Travel Medicine and Infectious Disease*	Muehlenbein, M. P., Martinez, L. A., Lemke, A. A., Ambu, L., Nathan, S., Alsisto, S., & Sakong, R. (2010). Unhealthy travelers present challenges to sustainable primate ecotourism. Travel medicine and infectious disease, 8(3), 169–175. https://doi.org/10.1016/j.tmaid.2010.03.004	n/a	Wild Animals (generally)
145	*Ursus*	Clark, J. D., Huber, D., & Servheen, C. (2002). Bear Reintroductions: Lessons and Challenges: Invited Paper. Ursus, 13, 335–345. http://www.jstor.org/stable/3873214	Bear, reestablishment, reintroduction, restoration, translocation, Ursus america	Bears
146	*Ursus*	Clark, J. E., Pelton, M. R., Wear, B. J., & Ratajczak, D. R. (2002). Survival of orphaned black bears released in the Smoky Mountains. Ursus, 269-273.	Black bear, days post-release, orphaned bears, rehabilitation, release, survival, Ursus	Bears
147	*Verge: Studies in Global Asias*	Choi, S. Y. (2023). Multispecies Justice beyond Animal Rights: Saebyeogi (새벽이) Sanctuary on Instagram. Verge: Studies in Global Asias, 9(2), 62-73.	n/a	Domesticated Animals
148	*Wildlife Research*	Palmer, N., Smith, M. J., Ruykys, L., Jackson, C., Volck, G., Riessen, N., ... & Palmer, B. (2020). Wild-born versus captive-bred: a comparison of survival and refuge selection by translocated numbats (Myrmecobius fasciatus). Wildlife Research, 47(3), 217-223.	conservation biology, population management, wildlife management	Marsupials (Numbats)
149	*Zoo Biology*	Curry, B. A., Drane, A. L., Atencia, R., Feltrer, Y., Howatson, G., Calvi, T., ... & Shave, R. (2023). Body mass and growth rates in captive chimpanzees (Pan troglodytes) cared for in African wildlife sanctuaries, zoological institutions, and research facilities. Zoo Biology, 42(1), 98-106.	growth, maturation, sexual dimorphism	Primates (Chimpanzees)
150	*Zoo Biology*	Hansen, B. K., Fultz, A. L., Hopper, L. M., & Ross, S. R. (2018). An evaluation of video cameras for collecting observational data on sanctuary-housed chimpanzees (Pan troglodytes). Zoo biology, 37(3), 156–161. https://doi.org/10.1002/zoo.21410	behavior; primate; technology; welfare	Primates (Chimpanzees)
